# Identification of macrophage activation-related biomarkers in obese type 2 diabetes that may be indicative of enhanced respiratory risk in COVID-19

**DOI:** 10.1038/s41598-021-85760-y

**Published:** 2021-03-19

**Authors:** Abu Saleh Md Moin, Thozhukat Sathyapalan, Ilhame Diboun, Stephen L. Atkin, Alexandra E. Butler

**Affiliations:** 1 Diabetes Research Center (DRC), Qatar Biomedical Research Institute (QBRI), Hamad Bin Khalifa University (HBKU), Qatar Foundation (QF), PO Box 34110, Doha, Qatar; 2 Academic Endocrinology, Diabetes and Metabolism, Hull York Medical School, Hull, UK; 3 Hamad Bin Khalifa University (HBKU), Doha, Qatar; 4 Royal College of Surgeons in Ireland Bahrain, Adliya, Kingdom of Bahrain

**Keywords:** Endocrinology, Endocrine system and metabolic diseases, Obesity

## Abstract

Hyperactivation of the immune system through obesity and diabetes may enhance infection severity complicated by Acute Respiratory Distress Syndrome (ARDS). The objective was to determine the circulatory biomarkers for macrophage activation at baseline and after serum glucose normalization in obese type 2 diabetes (OT2D) subjects. A case-controlled interventional pilot study in OT2D (n = 23) and control subjects (n = 23). OT2D subjects underwent hyperinsulinemic clamp to normalize serum glucose. Plasma macrophage-related proteins were determined using Slow Off-rate Modified Aptamer-scan plasma protein measurement at baseline (control and OT2D subjects) and after 1-h of insulin clamp (OT2D subjects only). Basal M1 macrophage activation was characterized by elevated levels of M1 macrophage-specific surface proteins, CD80 and CD38, and cytokines or chemokines (CXCL1, CXCL5, RANTES) released by activated M1 macrophages. Two potent M1 macrophage activation markers, CXCL9 and CXCL10, were decreased in OT2D. Activated M2 macrophages were characterized by elevated levels of plasma CD163, TFGβ-1, MMP7 and MMP9 in OT2D. Conventional mediators of both M1 and M2 macrophage activation markers (IFN-γ, IL-4, IL-13) were not altered. No changes were observed in plasma levels of M1/M2 macrophage activation markers in OT2D in response to acute normalization of glycemia. In the basal state, macrophage activation markers are elevated, and these reflect the expression of circulatory cytokines, chemokines, growth factors and matrix metalloproteinases in obese individuals with type 2 diabetes, that were not changed by glucose normalisation. These differences could potentially predispose diabetic individuals to increased infection severity complicated by ARDS.

*Clinical trial reg. no*: NCT03102801; registration date April 6, 2017.

## Introduction

Whether type 2 diabetes (T2D) increases risk for development of acute respiratory distress syndrome (ARDS) has been controversial^1,2^. Recently, SARS-CoV-2 infection leading to severe COVID-19 disease has been shown to reflect underlying health conditions^3^, diabetes being incontrovertibly associated with poor outcome in patients with COVID-19^4^. Obesity clearly represents a significant, independent risk factor, body mass index being positively correlated with risk of ARDS^5^. Therefore, the combination of obesity and T2D places patients at high risk for severe infection and poor outcomes.

Acute respiratory distress syndrome (ARDS) results from an excessive and uncontrolled systemic inflammatory response where distinct populations of macrophages, resident alveolar macrophages (AMs), and recruited macrophages from the blood undergo dramatic changes in number and phenotype, playing a causal role in pathogenesis and resolution of ARDS^6^. ARDS is the hallmark of severe COVID-19 disease, responsible for the increased mortality associated with SARS-CoV-2 infection particularly in diabetes^4^.

Macrophages are a heterogeneous population of innate immune cells that, especially in lung, serve as the first barriers of defense against extrinsic invaders and airborne particles^7^. Macrophages play a pivotal role in inflammatory processes, clearing cellular debris and effecting resolution post-inflammation. Macrophages undergo polarization in response to the extracellular environment^8,9^, including infection, leading to two polarization states: the classically activated phenotype (M1) and the alternatively activated phenotype (M2)^10^. M1 macrophages have pro-inflammatory and cytotoxic properties and play a key role in virus clearance. Conversely, M2 (anti-inflammation) cells play a key role in tissue remodeling and matrix deposition post-injury^11^.

Th1-derived interferon-gamma (IFN-γ) fights intracellular infection in acquired immunity, and several immunomodulatory agents like lipopolysaccharide (LPS) or other cytokines such as granulocyte–macrophage colony stimulating factor (GM-CSF) and tumor necrosis factor-alpha (TNF-α), are responsible for differentiation of classically activated M1 macrophages. Conversely, M2 macrophages are activated by Th2-type cytokines, including IL-4 and IL-13, macrophage colony-stimulating factor (M-CSF) or Toll-like receptor (TLR) ligands^12^. Transcriptional analysis of human alveolar macrophages revealed that M1 macrophages express CD69, CD38, Toll-like receptor 2 (TLR2), TLR4, CXC-chemokine ligand 9 (CXCL9), CXCL10 and CXCL11, whilst M2 macrophages express mannose receptors (CD206), matrix metalloproteinase 2 (MMP2), MMP7, MMP9, CD163 and arginase^13^. In steady state, no strict polarization pattern of macrophages (for example, alveolar macrophages) exists and they can adopt a hybrid (both M1 and M2) phenotype in healthy humans^14^. Prolonged M1 or M2 phenotypes are associated with non-healing chronic ARDS^15^.

An increase in tissue macrophages is a common feature of both macro and microvascular diabetic complications, including nephropathy, atherosclerosis, neuropathy, and retinopathy. Hyperglycemia is thought to depress phagocytic ability^16^; in vitro studies have shown that high glucose concentrations increased expression of certain M2 polarization markers such as MMP9 and CD169^17^.

Fibroproliferative lung alveolar macrophage-derived TGF-β1, PDGF-β, MMP7 and MMP9 are elevated in diabetes, leading to lung stiffening and making patients more vulnerable to COVID-19 disease^18^. Glucose variability has been associated with increased tissue damage through oxidative stress^19^ and increased glucose variability was related to more severe ARDS in COVID-19 disease^20^. Therefore, we hypothesized that in obese type 2 diabetes (OT2D), even in the basal state, macrophages are activated and thus impaired or aberrant macrophage protein expression is already present in OT2D in comparison to non-diabetic controls, that may increase both infection susceptibility and disease severity, predisposing patients with OT2D to ARDS that is the hallmark of severe COVID-19 disease. To address this question, we measured macrophage markers in plasma of OT2D versus non-diabetic subjects and determined whether alterations in levels of macrophage activation markers in OT2D could be altered by normalizing glucose levels with a hyperinsulinemic insulin clamp.

## Materials and methods

### Study design

A prospective parallel pilot study performed in the Diabetes Research Centre at Hull Royal Infirmary in adults with type 2 diabetes (n = 23) and controls without diabetes (n = 23) (Clinical trial reg. no: NCT03102801; registration date 06/04/2017; start date 01/03/2017, end date 10/01/2018) ^21^. Patient characteristics of T2D and control groups are shown in Supplementary Table 1. All participants provided written informed consent. The trial was approved by the North West-Greater Manchester East Research Ethics Committee (REC number: 16/NW/0518) and conducted according to the Declaration of Helsinki.

### Study participants

All participants were Caucasian, aged 40–70 years. The T2D group had been diagnosed for < 10 years; all were on a stable dose of medication (metformin, statin and/or angiotensin-converting enzyme inhibitor/angiotensin receptor blocker) for the preceding three months^21^. T2D subjects were excluded if on any anti-glycemic medication other than metformin or with poor glycemic control [HbA1c levels ≥ 10% (86 mmol/mol)]. Control subjects were excluded if diagnosed with type 1 or 2 diabetes or if HbA1c levels > 6% (42 mmol/mol). The following exclusion criteria were applied for both groups: current smokers, body mass index (BMI) < 18 or > 50 kg/m^2^, excessive alcohol consumption, renal or liver disease, history or presence of malignant neoplasms within the last 5-years, diagnosis of psychiatric illness, history of pancreatitis or gastrointestinal tract surgery.

### Hyperinsulinemic euglycemic clamp studies

Detailed procedures and patient prerequisites for the hyperinsulinemic euglycemic clamp procedure were described previously^22^. The insulin infusion rate was constant throughout the clamp [60 mU/body surface area(m^2^)/min], while the rate of 20% dextrose infusion was adjusted every 5-min to achieve target blood glucose level. Baseline glucose for OT2D was 7.6 ± 0.4 mmol/l (136.8 ± 7.2 mg/dl), reduced to 4.5 ± 0.07 mmol/l (81 ± 1.2 mg/dl) with the clamp for 1-h. For controls, blood glucose was maintained at 4.9 ± 0.1 mmol/l (88.2 ± 1.8 mg/dl).

### Blood processing and biochemical markers measurement

As previously described^22^, “blood samples were separated immediately by centrifugation at 2000 g for 15-min at 4 °C, and the aliquots were stored at − 80 °C, within 30-min of blood collection, until batch analysis. High sensitivity C-reactive protein (hsCRP) was measured using Synchron systems CRPH reagent kit (Beckman-Coulter, UK) per manufacturer’s protocol. Fasting plasma glucose (FPG) was measured using a Synchron LX 20 analyzer (Beckman-Coulter) per manufacturer’s recommended protocol.”

### SOMA-scan measurements

Plasma protein quantification was performed using a Slow Off-rate Modified Aptamer (SOMAmer)–based protein array, as previously described (version 3.1 of the SomaScan Assay)^22^. Briefly, EDTA plasma samples were diluted and the following assay steps were performed in sequence: binding, Catch 1, Cleave, Catch II, elution and quantification. Normalization of raw intensities, hybridization, median signal and calibration signal were performed based on the standard samples included on each plate, as previously described^23^. Plasma protein concentrations were expressed as relative flurescent unitis (RFU).

### Statistics

No studies detailing changes in macrophage-related proteins in response to hypoglycemia are available on which to base a power calculation. Sample size for pilot studies has been reviewed by Birkett and Day^24^. They concluded a minimum of 20 degrees-of-freedom was required to estimate effect size and variability. Hence, we needed to analyse samples from minimum 20 patients per group. Data trends were visually evaluated; non-parametric tests were applied on non-normal data using the Kolmogorov–Smirnov Test. Comparison between groups was performed using Student’s t-test. A *p*-value of < 0.05 was considered statistically significant. Statistical analysis was performed using Graphpad Prism (San Diego, CA, USA).

### Ethics approval and consent to participate

The trial was approved by the North West-Greater Manchester East Research Ethics Committee (REC number: 16/NW/0518). The Hull and East Yorkshire Hospitals NHS Trust sponsored this study. All patients gave written informed consent.

### Consent for publication

All authors gave their consent for publication.

## Results

### Pro-inflammatory stimuli for monocyte to macrophage differentiation are not altered in obese T2D

Plasma levels of IFN-γ were not different in OT2D versus controls (676 ± 62 vs 610 ± 20 relative fluorescent units [RFU] of IFN-γ, OT2D vs control, *p* = ns) (Supplementary Fig. 1A). Plasma levels of IL-4 and IL-13 were also not different in OT2D compared to control (294 ± 10 vs 278 ± 6 RFU of IL-4 and 598 ± 23 vs 663 ± 59 RFU of IL-13, OT2D vs control, *p* = ns) (Supplementary Fig. 1B and C). This data suggests that no increased Th1 or Th2-mediated immune response is present in obese OT2D subjects (basal condition).

### Altered plasma LPS (an activator of pro-inflammatory monocyte to M1 macrophage differentiation) levels in obese subjects with T2D (OT2D)

**Figure 1:**
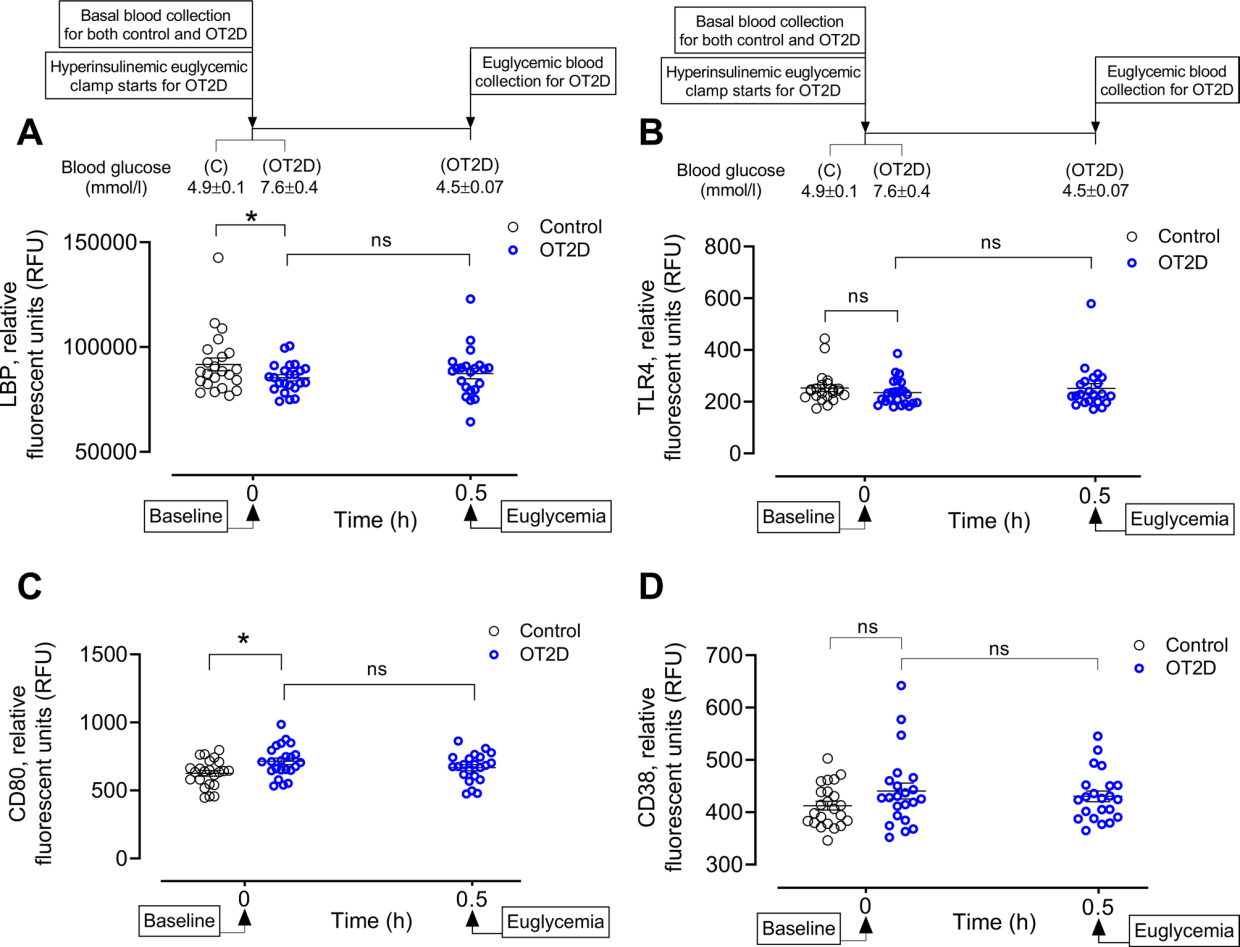
Circulatory levels of M1 macrophage activation markers in the steady state of obese type 2 diabetes (OT2D). Plasma LBP (**A**), TLR4 (**B**), CD80 (**C**) and CD38 (**D**) levels in control subjects (black circles) and obese subjects with T2D (blue circles). Basal level of plasma LBP was higher (85,311 ± 1453 vs 91,747 ± 3048 RFU of LBP, OT2D vs control, *p* < 0.05) but TLR4 was unchanged in OT2D (235 ± 11 vs 254 ± 13 RFU of TLR4, OT2D vs control, *p* = ns). Basal levels of plasma M1 macrophage surface markers CD38 and CD80 were also higher in OT2D (714 ± 24 vs 626 ± 21 RFU of CD80, OT2D vs control, *p* < 0.01; 441 ± 15 vs 408 ± 7 RFU of CD38, OT2D vs control, *p* < 0.05). Acute normalization of glycemia had no effect on levels of LBP, CD80 or CD38 in T2D subjects (A-D). Data were presented as mean ± SEM. *, *p* < 0.05, **, *p* < 0.01.

Classically activated M1 macrophages constitute the first line of defense against intracellular pathogens and therefore exhibit a high level of phagocytic activity. A significant reduction of plasma lipopolysaccharide binding protein (LBP) was found in OT2D versus controls (85,311 ± 1453 vs 91,747 ± 3048 RFU of LBP, OT2D vs control, *p* < 0.05) suggesting LPS-mediated activation of pro-inflammatory monocytes to M1 macrophages in OT2D (Fig. 1A). Further suggestion of LPS-induced activation of M1 macrophages in OT2D was shown by no change of plasma toll like receptor-4 (TLR4) levels in OT2D (235 ± 11 vs 254 ± 13 RFU of TLR4, OT2D vs control, *p* = ns) (Fig. 1B) that suggested elevated LPS-mediated endocytosis of TLR-4 in OT2D^25^.

One of the markers that best characterizes human M1-like macrophages activation in response to IFN-γ and ILPS is CD80^26^. Plasma CD80 was increased in T2D (714 ± 24 vs 626 ± 21 RFU of CD80, T2D vs control, *p*< 0.01) (Fig. 1C). In addition, the basal level of plasma CD38, another LPS-induced M1 macrophage marker was elevated in OT2D (441 ± 15 vs 408 ± 7 RFU of CD38, OT2D vs control, *p* < 0.05) (Fig. 1D). There were no changes in plasma levels of LBP, TLR4, CD80 and CD38 level in OT2D in response to insulin-induced acute normalization of glycemia (Fig. 1A–D).

### Pro-inflammatory cytokines associated with LPS-induced M1 macrophages in OT2D

**Figure 2:**
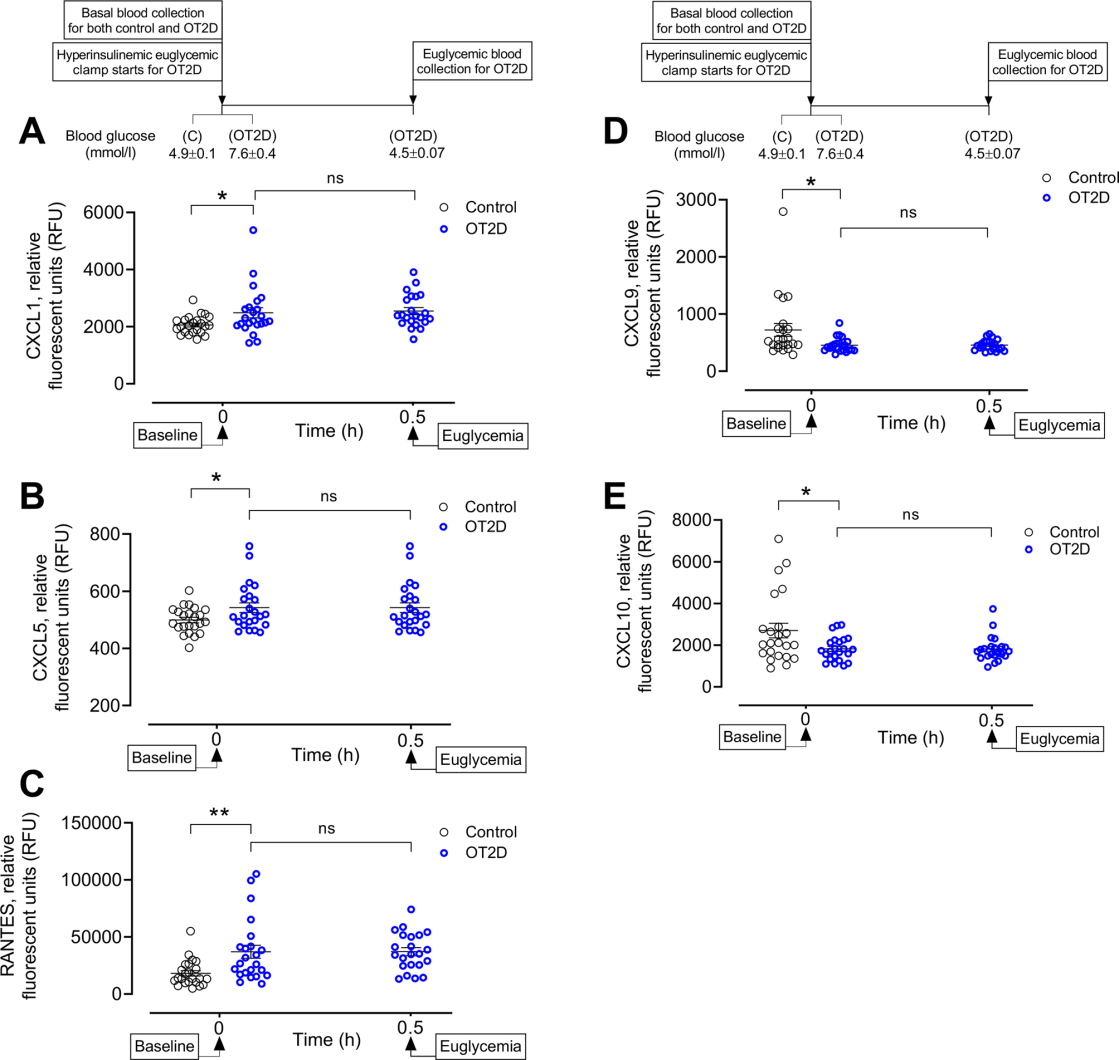
Circulatory levels of cytokines and chemokines released from activated M1 macrophages in the steady state of obese type 2 diabetes (OT2D). Plasma CXCL1 (**A**), CXCL5 (**B**), CCL5 (RANTES) (**C**), CXCL9 (**D**) and CXCL10 (**E**) levels in control subjects (black circles) and obese subjects with T2D (blue circles). Basal level of plasma CXCL1, CXCL5 and RANTES was higher in OT2D compared to control (2487 ± 177 vs 2060 ± 67 RFU of CXCL1, OT2D vs control, *p* < 0.05; 543 ± 17 vs 500 ± 10 RFU of CXCL5, OT2D vs control, *p* < 0.05; 36,961 ± 5692 vs 18,162 ± 2393 RFU of RANTES, OT2D vs control, *p* < 0.01). Basal levels of plasma CXCL9 and CXCL10 were lower in T2D compared to control (455 ± 26 vs 722 ± 113 RFU of CXCL9, and 1825 ± 122 vs 2706 ± 352 RFU of CXCL10, OT2D vs control, *p* < 0.05). Acute normalization of glycemia had no effect on levels of CXCL1. CXCL5, RANTES, CXCL9 and CXCL10 in T2D subjects (A-E). Data were presented as mean ± SEM. *, *p* < 0.05, **, *p* < 0.01.

To determine the biomarkers (pro-inflammatory cytokines and chemokines released by LPS-TLR4 interaction) of activated M1 macrophages, basal levels of cytokines TNF-α, IL-6, IL-1β, IL-12 and chemokines CXCL-1, CXCL3, CXCL5, CXCL8, CXCL9, CXCL10 were measured. Basal levels of TNF-α, IL-1β, IL-6 and IL-12 were not changed in OT2D subjects (419 ± 19 vs 423 ± 21 RFU of TNF-α; 959 ± 49 vs 971 ± 54 RFU of IL-1β; 192 ± 6 vs 208 ± 17 RFU of IL-6 and 244 ± 42 vs 196 vs 18 RFU of IL-12, OT2D vs control, *p* = ns) (Supplementary Fig. 2A–D). For chemokines, the basal levels of CXCL1 and CXCL5 were higher in OT2D (2487 ± 177 vs 2060 ± 67 RFU of CXCL1 and 543 ± 17 vs 500 ± 10, RFU of CXCL5, OT2D vs control, *p* < 0.05) (Fig. 2A–B). The level of CXCL3 and CXCL8 were not different in OT2D (377 ± 35 vs 370 ± 56 RFU of CXCL3; 543 ± 17 vs 499 ± 10 RFU of CXCL5 and 838 ± 33 vs 814 ± 67 RFU of CXCL8, OT2D vs control, *p* = ns) (Supplementary Fig. 2E–F). No difference in the basal level of plasma MFG-E8 level, associated with increased phagocytosis, in OT2D was found (1114 ± 68 vs 1216 ± 100 RFU of MGF-E8, OT2D vs control, *p* = ns) (Supplementary Fig. 2G). Two chemokines that have been shown to be increased in LPS-induced M1 polarized macrophages are CXCL-8 (IL-8) and RANTES (CCL5): basal IL-8 level did not differ in OT2D; however, plasma RANTES level was significantly higher (~ two-fold) in OT2D (36,961 ± 5692 vs 18,162 ± 2393 RFU of RANTES, OT2D vs control, *p* < 0.01) (Fig. 2C).

Measurement of two classically activated M1 macrophage markers, CXCL9 and CXCL10, showed a significant reduction of the basal level of plasma CXCL9 (455 ± 26 vs 722 ± 113 RFU of CXCL9, OT2D vs control, *p* < 0.05) and CXCL10 (1825 ± 121 vs 2706 ± 352 RFU of CXCL10, OT2D vs control, *p* < 0.05) in OT2D compared to control (Fig. 2D–E). Correction to normoglycemic did not alter the plasma levels of M1 macrophage activation markers in OT2D (Fig. 2A–E).

### Activation of anti-inflammatory subsets of monocytes to M2 macrophages in obese individuals with type 2 diabetes (OT2D).

**Figure 3:**
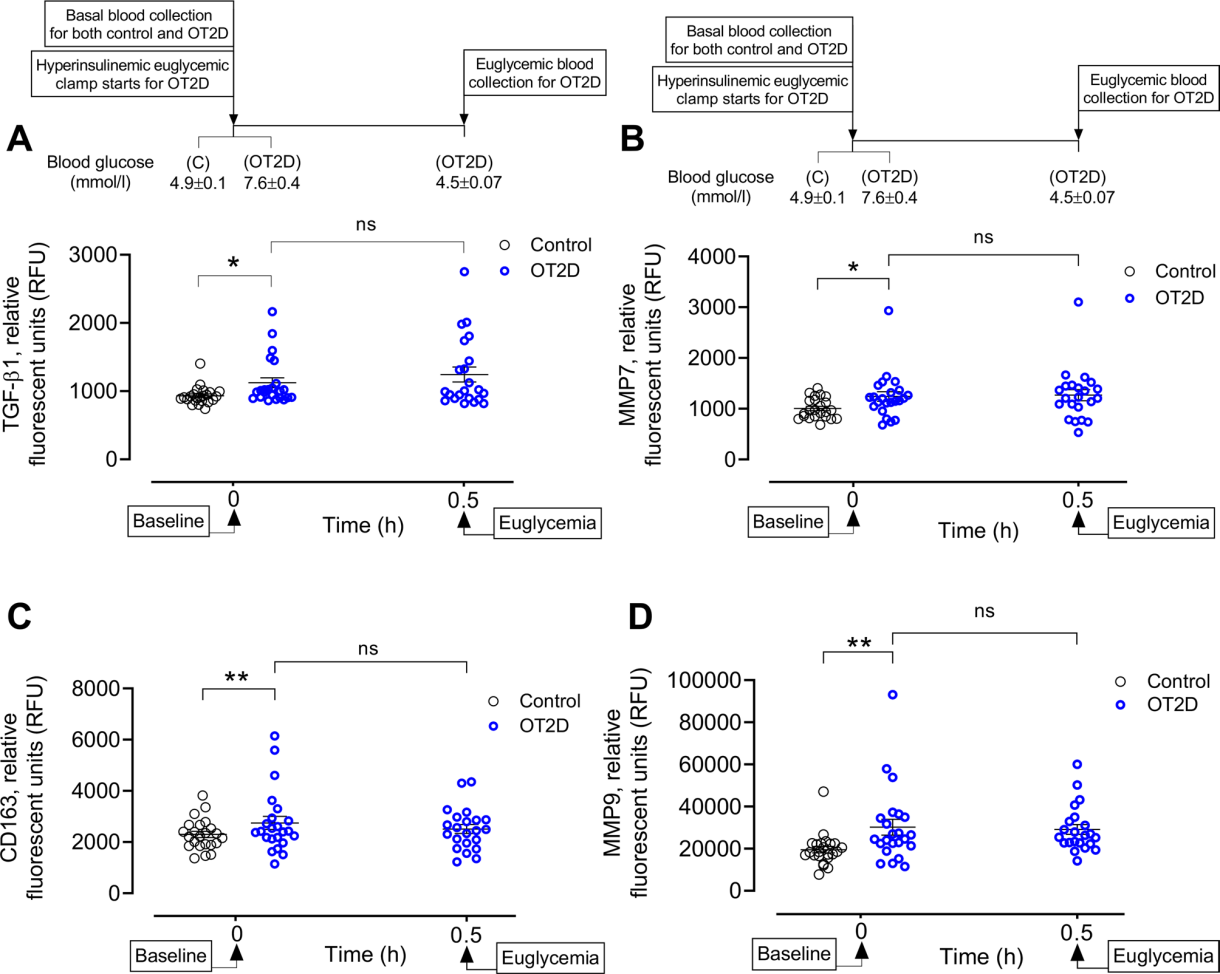
Circulatory levels of activated M2 macrophage markers in the steady state of obese type 2 diabetes (OT2D). Plasma TGF-β1 (**A**), CD163 (**B**), MMP7 (**C**) and MMP9 (**D**) levels in control subjects (black circles) and obese subjects with T2D (blue circles). Basal levels of plasma TGF-β1, CD163, MMP7 and MMP9 were higher in OT2D compared to control (1123 ± 72 vs 933 ± 27 RFU of TGF-β1, OT2D vs control, *p* < 0.01; 2748 ± 256 vs 2297 ± 124 RFU of CD163, OT2D vs control, *p* < 0.05). Two matrix metalloproteinases MMP7 and MMP9 were also increased in OT2D (1242 ± 93 vs 1005 ± 42 RFU of MMP7, OT2D vs control, *p* < 0.05 and 30,193 ± 3746 vs 19,532 ± 1562 RFU of MMP9, OT2D vs control, *p* < 0.01). Acute normalization of glycemia had no effect on levels of LBP, CD80 or CD38 in T2D subjects (A-D). Data were presented as mean ± SEM. *, *p* < 0.05, **, *p* < 0.01.

With the findings described above showing no change in basal level of plasma IL-4/IL-3 but likely elevated LPS level in OT2D, we hypothesized that anti-inflammatory subsets of circulatory monocytes are activated to M2 macrophages in OT2D, and the activation is mediated either by LPS (M2b) or TGB-β (M2c). Consistent with our hypothesis, we found a significant elevation of basal TGF-β1 level in OT2D (1123 ± 72 vs 933 ± 27 RFU of TGF-β1, OT2D vs control, *p* < 0.01) (Fig. 3A) [recently reported in^18^]. Basal level of plasma CD163 (soluble CD163, sCD163) was higher in OT2D compared to control (2748 ± 256 vs 2297 ± 124 RFU of sCD163, OT2D vs control, *p* < 0.05) (Fig. 3B). We further measured the plasma CD206 (soluble CD206, sCD206), another potent marker for M2 macrophage receptor, and found that there was no change in the basal level of plasma CD206 (10,600 ± 477 vs 9885 ± 431 RFU of sCD206, OT2D vs control, *p* = ns) (Supplementary Fig. 3A).

We next explored other potent M2 macrophage soluble receptor markers to determine if the basal concentration was also increased in the plasma of OT2D compared to control subjects. Our data indicate that there were no changes in plasma arginase and CD200R1 (888.2 ± 45.3 vs 963.1 ± 97.2 RFU of arginase and 1055.6 ± 41.4 vs 1102.8 ± 40.6 RFU of CD200R1, OT2D vs control, *p* = ns) (Supplementary Fig. 3B and C) in OT2D compared to control. Interestingly, the level of plasma CD200 was downregulated in OT2D (2241 ± 85 vs 2426 ± 67 RFU of CD200, OT2D vs control, *p* < 0.05) (Supplementary Fig. 3D). Basal levels of dendritic cell specific ICAM-3 grabbing nonintegrin (DC-SIGN, also known as CD209) and CD36 were significantly lower in OT2D compared to control (1221 ± 63 vs 1440 ± 102 RFU of CD209 and 9752 ± 614 vs 11,360 ± 627 RFU of CD36, OT2D vs control, *p* < 0.05) (Supplementary Fig. 3E and F).

The matrix metalloproteinases (MMPs) MMP7 and MMP9 were found to be elevated in OT2D compared to control (1242 ± 93 vs 1005 ± 42 RFU of MMP7 and 30,193 ± 3746 vs 19,532 ± 1562 RFU of MMP9, OT2D vs control, *p* < 0.05) (Fig. 3C–D) [recently reported in^18^]. Correction to normoglycemic had no effect on plasma levels of M2 macrophage activation markers in OT2D (Fig. 3A–D).

### Soluble markers related to adipose tissue macrophage (ATM) activation in OT2D

**Figure 4:**
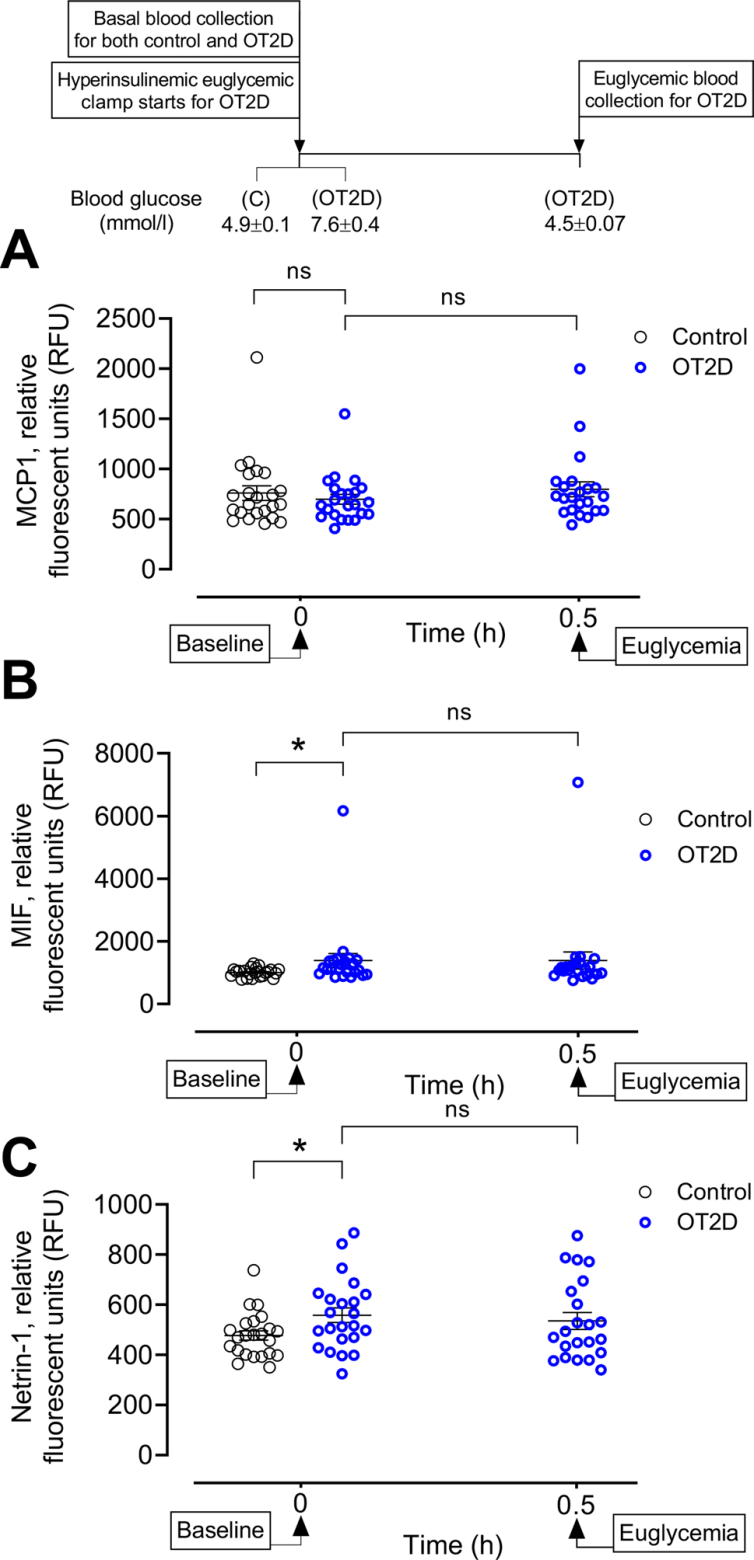
Circulatory levels of adipose tissue macrophage (ATM) markers in the steady state of obese type 2 diabetes (OT2D). Plasma level of MCP-1 (**A**), MIF (**B**) and netrin-1 (**C**) in control subjects (black circles) and obese subjects with T2D (blue circles). Basal level of plasma MCP-1 did not alter in OT2D compared to control (698 ± 49 vs 760 ± 73 RFU of MCP-1, OT2D vs control, *p* = ns). Basal level of plasma MIF and netrin-1was increased in OT2D (1387 ± 223 vs 1007 ± 29 RFU of MIF-1 and 558 ± 30 vs 478 ± 19 RFU of netrin-1, OT2D vs control, *p* < 0.05). Acute normalization of glycemia had no effect on levels of LBP, CD80 or CD38 in T2D subjects (A-C). Data were presented as mean ± SEM. *, *p* < 0.05.

Since the T2D subjects in this study were obese, we sought to determine the circulatory markers for activation of adipose tissue macrophages (ATMs) in OT2D. Human ATMs are characterized by their expression of CD163 and we showed in the previous section that the plasma CD163 level was increased in OT2D, suggesting activation of ATMs in OT2D. To determine if those ATMs are derived from circulating monocytes, we measured the monocyte chemoattractant protein-1 (MCP-1, also known as CCL2). Our data indicated that circulatory MCP-1 level did not differ in OT2D compared to control (698 ± 49 vs 760 ± 73 RFU of MCP-1, OT2D vs control, *p* = ns) (Fig. 4A), suggesting reduced or no migration of monocyte-derived macrophages in adipose tissue. This observation was supported by the level of macrophage migration inhibitory factor (MIF) which was higher in OT2D (1387 ± 223 vs 1007 ± 29 RFU of MIF-1, OT2D vs control, *p* < 0.05) (Fig. 4B). We further measured the level of netrin-1 to determine if the ATM activation is mediated by a local modulator. Plasma netrin-1 level was higher in OT2D (558 ± 30 vs 477 ± 19 RFU of netrin-1, OT2D vs control, *p* < 0.05) (Fig. 4C)***.*** No changes were observed in the plasma levels of adipose tissue macrophages in response to acute normalization of glycemia in OT2D (Fig. 4A–C).

### Association of macrophage-related proteins with BMI

Since the T2D subjects in this study were obese, we next considered which of the macrophage-associated circulatory proteins associated with BMI. Six proteins showed an association with BMI: RANTES (*p* = 0.002), CXCL1 (*p* = 0.002), MIF (*p* = 0.03), LBP (*p* = 0.03), IL13 (*p* = 0.03) and CD200R1 (*p* = 0.03). Whilst the above proteins associated with BMI, suggesting that BMI may serve as a surrogate for insulin resistance or increased adipokine activity, other proteins were BMI-independent, namely CD80 (*p* = 0.004), CXCL10 (*p* = 0.012), TGF-β1 (*p* = 0.02), and MMP9 (*p* = 0.03), indicating that these BMI-independent proteins may relate specifically to T2D.

## Discussion

This study describes the novel finding of large-scale circulatory markers that are responsible for the differentiation of both pro and anti-inflammatory monocytes to M1 and M2 macrophages in OT2D and these circulatory markers were unaffected by acute normalization of glycemia. Our data demonstrate that, in the basal state, classical and alternative activation of macrophages in OT2D were not mediated by conventional mediators (IL-4, IL-13 or IFN-γ); rather, they may be activated by elevated levels of LPS. Increased markers of adipose tissue macrophage activation in OT2D support the model of adipocyte-macrophage interaction and metabolic endotoxemia-induced development of obesity and insulin resistance^27^. The circulatory cytokine and chemokine profiles in response to pro-inflammatory monocyte-derived M1 macrophage activation showed a unique pattern in OT2D. Most of the conventional M1 macrophage activation markers (mainly reported in animals) were not elevated in OT2D. However, we found high plasma levels of LPS-induced human M1 surface markers CD80, CD38 and pro-inflammatory chemokines CXCL1, CXCL5 and CCL5, that are released from activated M1 macrophages in OT2D. Our data also revealed that certain subclasses of anti-inflammatory monocytes differentiated to M2 macrophages that are induced by LPS or TGF-β1 are also activated in OT2D. The elevated levels of M2 activation markers TGF-β1, MMP7 and MMP9, led us to speculate that in certain tissues (e.g. lungs), M2 macrophages are abundant even in the basal state in OT2D. Elevated macrophage activation markers in OT2D were unchanged by acute insulin-induced euglycemia.

### LPS may be a mediator of pro- or anti-inflammatory monocyte-derived macrophage activation in obese type 2 diabetes

Since our study involved measuring only plasma proteins, we utilized surrogate markers of LPS elevation and its role in differentiation of subsets of pro-inflammatory monocytes to M1 macrophages in OT2D. We measured the level of plasma LPS-binding protein (LBP) as a determinant of LPS activity in our study subjects and found the LBP level was significantly lower in OT2D compared to controls. Previous publications have reported raised levels of LBP in obese subjects^28,29^, which is discrepant with our observations. The difference might be a consequence of case selection, as the obese subjects were smokers and consumed alcohol in both of those referenced reports. It is known that, in smokers, LBP levels are raised in bronchoalveolar lavage fluid (BALF)^30^; this is also the case in heavy drinkers, likely due to injury inflicted upon the gastrointestinal barrier by alcohol^31^. By contrast, in our study, smoking and alcohol consumption were exclusion criteria. Furthermore, LBP has a dual role in effecting LPS-induced macrophage activation that is concentration dependent; low concentrations of LBP promote LPS-induced activation of mononuclear cells (MNC), while high concentrations inhibit LPS-induced cellular stimulation^32^. Moreover, LBP binds to host cells and is internalized, and in the cytoplasm colocalizes with LPS^33^. Therefore, decreased LBP levels are reflective of raised LPS levels in the OT2D subjects reported here. The elevated LPS-induced activation of M1 macrophages in OT2D was further confirmed by plasma TLR4 levels that showed no difference between OT2D and controls, suggesting elevated LPS-mediated endocytosis of TLR4^25^.

None of the pro-inflammatory cytokines involved in either M1 or M2 macrophage activation, IFN-γ, IL- 4 and IL-13, were increased in OT2D, suggesting that there was no increase of Th1 or Th2-mediated responses in those OT2D subjects. One explanation might be that there were no inflammatory reactions triggered by an invading parasite or allergen in those OT2D cases, as there was no change in plasma CRP in OT2D (Supplementary Table 1).

### Pro-inflammatory monocyte-derived M1 macrophage activation in obese type 2 diabetes

Elevated CD38 and CD80 levels were found in OT2D, suggesting that LPS-induced activation of pro-inflamatory monocyte-derived M1 macrophages is induced in OT2D. Previous studies suggest CD38 and CD80 are the markers that best characterize LPS-induced human M1-like macrophage activation^26,34^, and CD80 has been reported as a co-stimulatory signaling molecule in alveolar macrophages^35^. However, pro-inflammatory cytokines and chemokines released by LPS-TLR4 interaction from M1 macrophage such as MFG-E8 expression were unchanged, suggesting that less mobilization of neutrophils occurs in response to LPS-induced M1 macrophage activation in OT2D.

Two chemokines, IL-8 and RANTES (CCL5), are released from LPS-induced M1 polarized macrophages^26^. We did not find any differences in basal IL-8 level in OT2D; however, plasma RANTES level was significantly (~ twofold) higher in OT2D, suggesting RANTES is a unique M1 macrophage activation marker for obese individuals with T2D. This is consistent with a previous report where RANTES (CCL5) was associated with LPS-induced M1 macrophage polarization^36^. Moreover, a transcriptional analysis of human alveolar macrophages that were polarized ex vivo using interferon-γ (IFN-γ), revealed the association of RANTES with M1 polarization^37^. Therefore, our data suggests lung alveolar macrophages as a possible tissue source of RANTES in OT2D.

Two potent chemokines, CXCL9 and CXCL10 that are markers of IFN-γ-stimulated classical M1 macrophages, were significantly reduced in our study subjects. This data is paradoxical to a previous report where LPS-induced elevation of serum CXCL9 and CXCL10 was reported in an LPS-induced ARDS model^38^; however, neutralization of IFN-γ showed a marked decrease of plasma CXCL9 and CXCL10 levels^39^, suggesting a relationship between stable IFN-γ levels and reduced levels of CXCL9 or CXCL10 in OT2D.

### Anti-inflammatory monocyte-derived M2 macrophage activation

Our data indicated that macrophages that are differentiated from anti-inflammatory monocytes (M2 macrophages) were activated, though there was no change in the basal level of plasma IL-4/IL-3; we therefore hypothesized that M2 macrophages in OT2D are activated either by LPS (M2b) or TGF-β (M2c). Consistent with our hypothesis, we found a significant elevation of basal TGF-β1 in OT2D, suggesting M2c is activated in OT2D. M2 macrophage activation was further confirmed by soluble CD163 (sDC13) (Hemoglobin-Haptoglobin Scavenger Receptor) expression that was higher in OT2D. CD163 is an M2 macrophage receptor, and its expression is amplified by IL-10, IL-6, M-CSF and glucocorticoids, while TNF-α, IFN-γ, LPS and TGF-β reduce its expression^40^. Our data, therefore, suggest a paradoxical effect of cytokines to stimulate the soluble CD163 level in OT2D; elevated circulating CD163 has been reported in obesity and T2D^41^.

The level of its ligand CD200 was downregulated in OT2D, though CD200R1, a surface marker of M2 macrophages, did not alter. Lung alveolar macrophages express high levels of CD200R at the basal condition and are upregulated during viral infection. Binding of CD200 (which is expressed on the luminal aspect of the airway epithelium) with CD200R imparts a unidirectional negative signal for resolution of inflammation to the lung alveolar macrophages^42^. Therefore, it is likely that an impaired resolution system after lung inflammation is present in OT2D. Two important M2 macrophage receptor markers, dendritic cell specific ICAM-3 grabbing nonintegrin (DC-SIGN, also known as CD209) and CD36, were significantly lower in OT2D. CD209 expression is enhanced by IL-4 but its effect is diminished by IFN-γ and TGF-β^43^. So, it is likely that elevated levels of TGF-β1 might play a role in the reduction of CD209 levels that have been related to hyperglycemia.

Two matrix metalloproteinases (MMPs), MMP7 and MMP9, were elevated in OT2D and transcriptional analyses revealed that both are expressed in human monocyte derived M2c macrophages^44^ or human alveolar M2 macrophages^45^, suggesting the possible activation of human alveolar M2 macrophages in OT2D. LPS induces the expression of both MMP7 and MMP9 in monocyte-derived macrophages^46^; therefore, our data strongly suggests that the circulating matrix metalloproteinases MMP7 and MMP9 represent macrophage activation markers in response to LPS stimulus in OT2D. Elevated M2 macrophage markers (especially, TGF-β1, MMP7 and MMP9) indicate that lung alveolar macrophages are activated. In COVID19, the pathogenesis of severe acute respiratory distress syndrome (ARDS) includes pulmonary fibrosis and edema. The major cellular sources of TGF-β in pulmonary fibrosis have been shown to be alveolar macrophages and metaplastic type II alveolar epithelial cells^47^. Elevated TGF-β1 in OT2D may predispose alveoli in a pre-fibrotic condition following SARS-CoV-2 infection and may be activated by MMP9^48^. MMP7 has also been reported as a potential peripheral blood biomarker of idiopathic pulmonary fibrosis^49^. Therefore, it is likely that, in OT2D, the lung epithelial barrier integrity is destabilized in response to the fibroproliferative activity of elevated TGF-β1, MMP7 or MMP9.

We normalized the prevailing hyperglycemia in OT2D subjects using an insulin clamp to investigate whether normalization of glycemia normalized the elevated macrophage markers. Acute normalization, however, did not have any effect upon basal macrophage activation in OT2D. This data is consistent with the LANCET trial, where treatment with insulin versus placebo or metformin did not reduce inflammatory biomarker levels despite improving glucose control^50^. However, increased glucose variability has been associated with increased tissue damage in diabetes, perhaps through oxidative stress mechanisms^19^ and was associated with more severe ARDS in SARS-CoV-2 infection^20^.

### Possible shedding mechanism of macrophage receptors in obese type 2 diabetes

Shedding of macrophage scavenger receptors is mediated by proteinase-dependent cleavage of the ectodomain of surface receptors. For example, there is considerable evidence supporting CD163 shedding as a matrix metalloproteinase (MMP)-dependent process^51^, especially as a positive correlation of sCD163 with MMP-9 levels in plasma was detected in healthy humans^52^. We also found that plasma MMP-9 levels were increased in obese T2D patients, suggesting MMP-mediated shedding of macrophage scavenger receptors in OT2D. In addition, ADAM17 (ADAM Metallopeptidase Domain 17)/TACE (tumor necrosis factor-α converting enzyme) is the key molecule that, in response to proinflammatory stimuli such as LPS, is activated and leads to cleavage of membrane-bound CD163 in its juxtamembrane region^53^. In the SOMA scan protein panels, ADAM17 was not included; however, we measured plasma levels of TIMP3, the tissue inhibitor of ADAM17^54^. Basal plasma TIMP3 levels were significantly higher in OT2D compared to control (Supplementary Fig. 2H). The increased levels of both circulating TIMP3 and MMP9 levels may restrict the tissue availability of active TIMP3 to inhibit ADAM17, as it has been proposed that, under certain pathological conditions, MMP expression is increased in response to elevated TIMP to promote the inhibitory binding of TIMPs to MMPs, and thus depleting the levels of unbound TIMP available for TIMP receptor-mediated signaling^55^. Therefore, it is likely that tissue ADAM17 remains hyper-activated to shed more CD163 in OT2D. Furthermore, macrophage scavenger receptor CD163 or CD206 shedding occurs in response to endotoxin lipopolysaccharide (LPS) from human monocytes and macrophages^56^. In our study, we demonstrated an elevated plasma LPS level even in the steady state of inflammation, suggesting LPS-mediated shedding of macrophage surface markers in OT2D.

### Strengths and limitations of the current study

Strengths of this study include inclusion of type 2 diabetic subjects with relatively short duration of disease who were relatively treatment naïve; the results may, however, be generalizable to other T2D cohorts. The major limitation of this study is the small numbers in each cohort; with a larger population, even greater differences in macrophage-related protein concentrations may have been discerned. Further limitations are that normalization of blood glucose was a single event and experimental glucose fluctuations clamped over a timecourse would have been more robust, and that plasma levels of these proteins may not be reflective of tissue concentrations. Another key limitation of our current study is that macrophage differentiation from monocytes occurs in tissues, with concomitant acquisition of a functional phenotype, in response to microenvironmental signals^57^ and all the soluble macrophage markers described here are also expressed in other tissues and may be activated in response to different stimuli. For example, CD38 is a multifunctional, membrane-associated ectozyme belonging to the ribosyl cyclase family and was initially discovered on the surface of thymocytes and T cells. Subsequently, it was found to be expressed by other types of immune cells and certain non-lymphoid tissues, including brain, eye, gut and prostate tissues^58^. Likewise, CD36 is also not solely a macrophage marker but is a membrane glycoprotein present on platelets, mononuclear phagocytes, adipocytes, hepatocytes, myocytes, and some epithelia. On microvascular endothelial cells, CD36 is a receptor for thrombospondin-1 and related proteins and functions as a negative regulator of angiogenesis^59^. In addition to these, CD163, described here as an M2 macrophage marker, is also expressed by other tissue resident macrophages. For example, in humans, CD163 expression is also restricted to the monocytic–macrophage lineage with high expression in, for example, red pulp macrophages, bone marrow macrophages, liver macrophages (Kupffer cells), lung macrophages, and in macrophages of several other tissues^60^.

In addition to these, a further limitation is that measuring the soluble surface markers of macrophages is not sufficient to determine the metabolic and inflammatory profile of macrophages^9,61–63^, which can be achieved through flow cytometry of these markers on T2D patient-derived altered monocytes or even the influence of this alteration in the differentiation of monocytes to macrophages in vitro. However, in our current study, it was beyond our scope to identify or categorize all the tissue sources of the activated macrophage markers. Moreover, the categorization of macrophages into the M1 (classical activation) and M2 (alternative activation) subtypes, is a simplified operation-based classification. Tissue macrophages have several distinct phenotypes, depending on specific signals from the microenvironment, location, and disease state. Therefore, to simplify our findings in such a large-scale soluble macrophage marker in obese type 2 diabetes, we categorized them as M1 or M2 macrophage markers.

## Conclusion

In conclusion, in the basal state, there are differences in macrophage activation markers that reflect circulatory cytokines, chemokines, growth factors and matrix metalloproteinases expression in obese individuals with type 2 diabetes that were not reversed by acute normalization of glycemia. These differences could potentially predispose diabetic patients to increased susceptibility to insult-induced ARDS and increased vulnerability for development of severe COVID-19 disease due to the enhanced tissue response to infection. Targeting TGF-β1, MMP7, or MMP9 might be considered as novel therapeutic options for treatment and/or prevention of ARDS as occurs in COVID-19 disease.

## Supplementary Information

Supplementary Information 1.

Supplementary Information 2.

Supplementary Information 3.

Supplementary Information 4.

## Data Availability

All the data for this study will be made available upon reasonable request to the corresponding author.
