# Supplementary material for: Identification of macrophage activation-related biomarkers in obese type 2 diabetes that may be indicative of enhanced respiratory risk in COVID-19
Source: Sci Rep. 2021 Mar 19;11:6428. doi: 10.1038/s41598-021-85760-y (PMC7979696; doi:10.1038/s41598-021-85760-y)
Supplement: Supplementary file 1 — Supplementary Information 1. [file 41598_2021_85760_MOESM1_ESM.docx]

**Supplementary table 1.** Demographic and clinical characteristics of the study participants.

Data is presented as Mean ± 1 SD.

| **Baseline** | **Type 2 Diabetes (n=23)** | **Controls (n=23)** | **p-value** |
| --- | --- | --- | --- |
| Age (years) | 64±8 | 60±10 | <0.0001 |
| Sex (M/F) | 12/11 | 11/12 | 0.77 |
| Weight (kg) | 90.9±11.1 | 79.5±8.8 | <0.0001 |
| Height (cm) | 167±14 | 169±5 | 0.64 |
| BMI (kg/m^2^) | 32±4 | 28±3 | <0.0001 |
| Systolic BP (mmHg) | 132±8 | 122±8 | 0.001 |
| Diastolic BP (mmHg) | 81±7 | 75±6 | 0.003 |
| Duration of diabetes (years) | 4.5±2.2 | N/A |  |
| HbA1c (mmol/mol) | 51.2±11.4 | 37.2±2.2 | <0.0001 |
| HbA1c (%) | 6.8±1.0 | 5.6±0.2 | <0.0001 |
| Total cholesterol (mmol/l) | 4.2±1.01.0 | 4.8±0.77 | 0.014 |
| Triglyceride (mmol/l) | 1.7±0.7 | 1.34±0.6 | 0.055 |
| HDL-cholesterol (mmol/l) | 1.1±0.3 | 1.5±0.4 | 0.001 |
| LDL-cholesterol (mmol/l) | 2.23±0.8 | 2.7±0.87 | 0.051 |
| CRP (mg/l) | 3.10±2.87 | 5.30±1110.03 | 0.66 |

BMI: Body mass index, BP: Blood pressure, HDL-cholesterol: High density lipoprotein cholesterol, LDL-cholesterol: Low density lipoprotein cholesterol, CRP: C-reactive protein. HbA1c: Haemoglobin A1c
