# Supplementary figures and images for: Identification of macrophage activation-related biomarkers in obese type 2 diabetes that may be indicative of enhanced respiratory risk in COVID-19
Source: Sci Rep. 2021 Mar 19;11:6428. doi: 10.1038/s41598-021-85760-y (PMC7979696; doi:10.1038/s41598-021-85760-y)

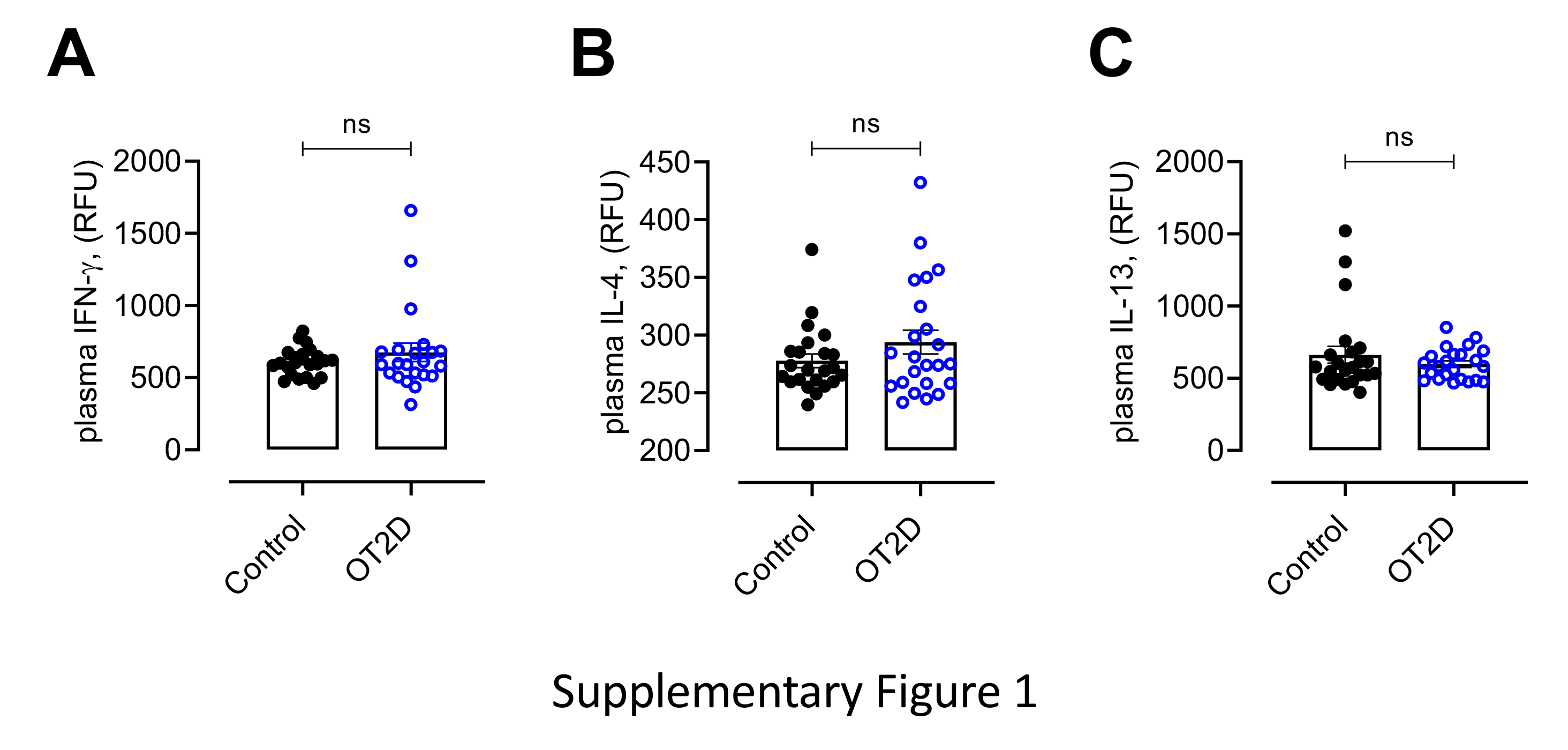

Supplement: Supplementary file 2 — Supplementary Information 2. [file 41598_2021_85760_MOESM2_ESM.tif]

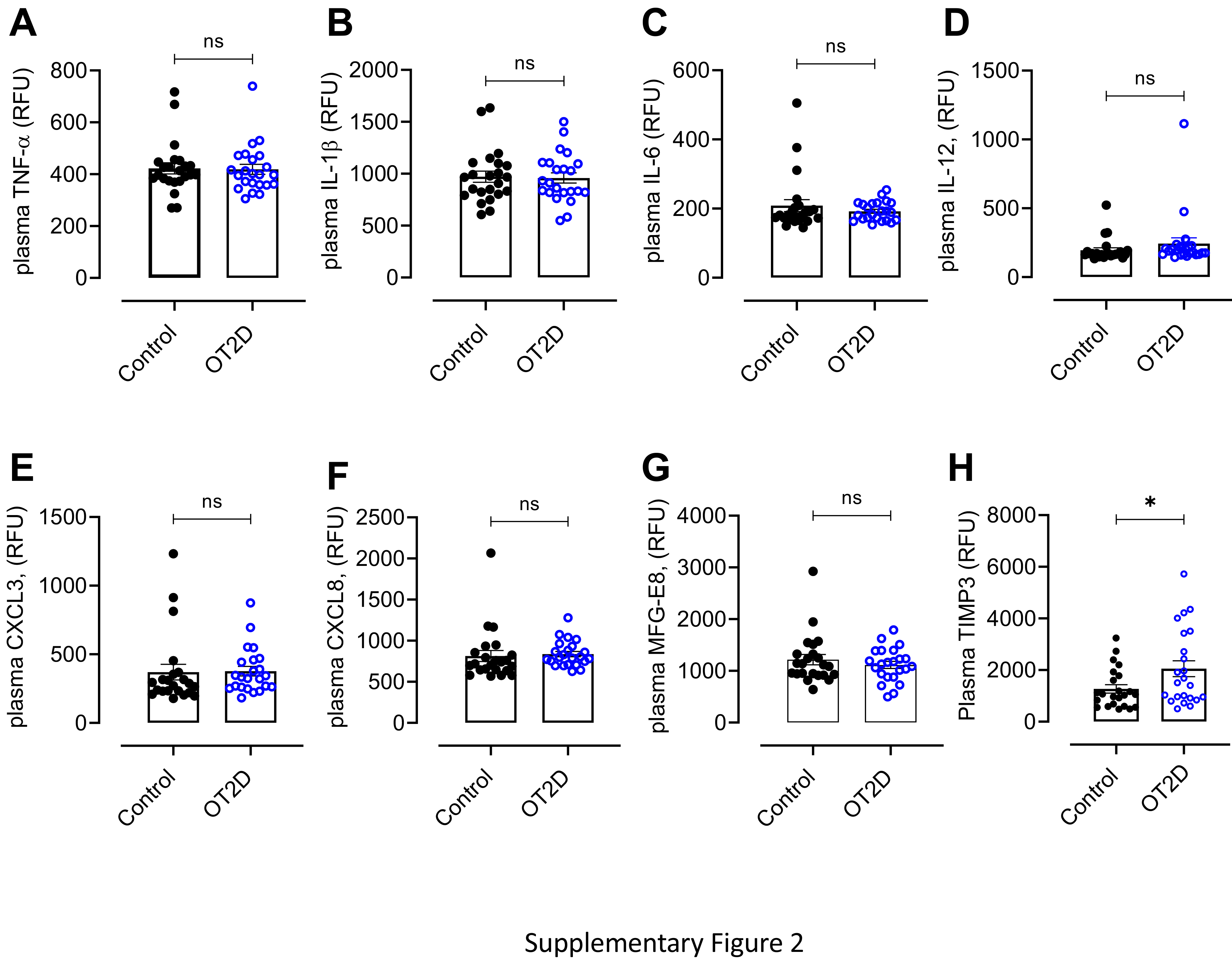

Supplement: Supplementary file 3 — Supplementary Information 3. [file 41598_2021_85760_MOESM3_ESM.tif]

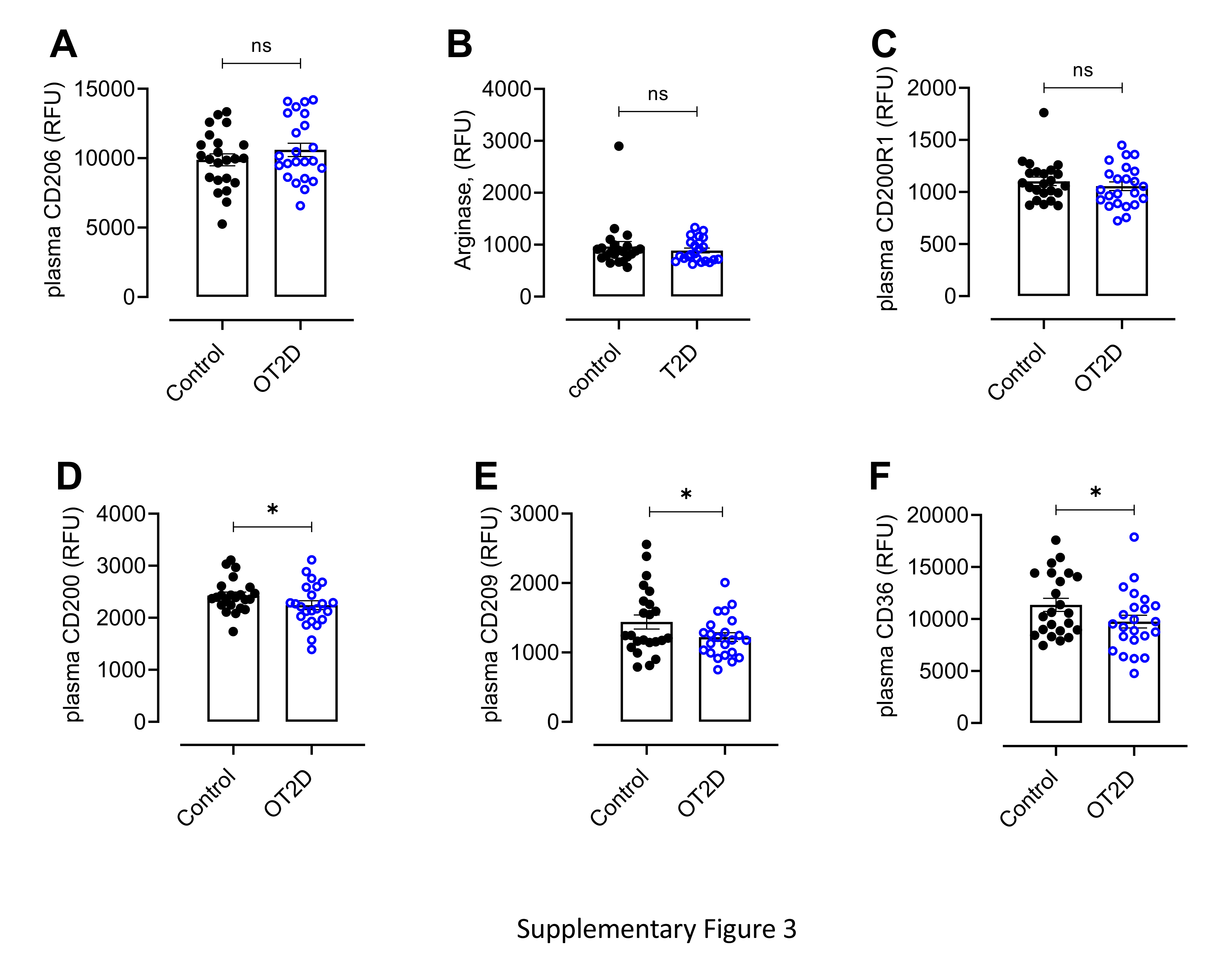

Supplement: Supplementary file 4 — Supplementary Information 4. [file 41598_2021_85760_MOESM4_ESM.tif]
